# 液体活检在肺癌伴恶性胸腔积液诊疗中的研究进展

**DOI:** 10.3779/j.issn.1009-3419.2021.101.24

**Published:** 2021-09-20

**Authors:** 灏 曾, 攀文 田, 为民 李

**Affiliations:** 610041 成都，四川大学华西医院呼吸与危重症医学科 Department of Respiratory and Critical Care Medicine, West China Hospital, Sichuan University, Chengdu 610041, China

**Keywords:** 液体活检, 肺肿瘤, 恶性胸腔积液, Liquid biopsy, Lung neoplasms, Malignant pleural effusion

## Abstract

肺癌是全球范围内死亡率最高的恶性肿瘤。晚期肺癌所致的恶性胸腔积液（malignant pleural effusion, MPE）严重影响患者的生活质量和预后。针对肿瘤的基因检测是制定精准治疗决策的基础，而组织活检取材存在风险，难以反复多次取样。液体活检由于具有无创性和可以反映肿瘤基因全貌的特点，在肺癌诊疗中的应用日益增多。基于血液样本的液体活检由于循环肿瘤DNA的含量极低，灵敏度和特异度有限。MPE中富含肿瘤细胞，检测其中的细胞游离DNA、细胞外囊泡和微小RNA等将有助于肺癌的诊断、治疗及预后的评估。本综述将探讨胸腔积液作为活检样本的研究，阐述液体活检在肺癌伴MPE诊疗中的研究进展。

## Abstract (en)

Lung cancer is the malignancy with the highest mortality rate worldwide. Malignant pleural effusion (MPE) caused by advanced lung cancer severely affects the quality of life and prognosis of patients. Tumor-associated genetic testing is the basis for making precise treatment decisions. There are some risks of tissue biopsy, and it is difficult to sample repeatedly. Due to its non-invasive and can reflect the full profile of tumor gene characteristics, liquid biopsy is increasingly used in the diagnosis and treatment of lung cancer. Because of the extremely low DNA level of circulating tumor, the sensitivity and specificity of liquid biopsy based on blood samples are limited. Tumor cells is enriched in MPE. The detection of cell-free DNA, extracellular vesicles and microRNA in MPE will be helpful to the diagnose, treatment and assess prognosis of patients with lung cancer. This review aims to discuss the research progress of liquid biopsy based on MPE in the diagnosis and treatment of lung cancer patients.

2020年全球癌症统计数据显示，肺癌作为第二常见的恶性肿瘤，在全球范围内，仍是死亡率最高的恶性肿瘤。在中国，肺癌发病率和死亡率居所有恶性肿瘤之首。2020年，中国新增肺癌患者达82万，死亡人数占全球肺癌死亡人数的40%^[1, 2]^。晚期肺癌患者的5年生存率不到15%，而Ia期肺癌患者的5年生存率可达80%-90%^[3, 4]^。可见，肺癌的早期筛查和早期诊断对提高患者生存率至关重要。

临床上常用于筛查和诊断肺癌的工具包括胸部X线、计算机断层扫描（computed tomography, CT）、低剂量CT以及正电子发射断层扫描（positron emission tomography, PET）等^[5, 6]^。胸部X线检查敏感性差，胸部CT能发现肺部微小结节，但是难以区分炎性结节和早期肺癌，而动态持续影像学监测可能会导致暴露于辐射下的患者引起辐射诱发的癌症^[7-9]^。确诊肺癌需要组织活检，但其为有创操作，存在一定风险，难以反复多次取样，无法实时监测肿瘤基因突变及耐药。此外，由于肿瘤具有异质性，局部组织的取样可能无法反映肿瘤基因全貌。基因组学技术的进展使液体活检应用于肿瘤的诊断、个体化治疗和预后评估成为可能^[10]^。

液体活检是伴随精准医学应运而生的一种新型诊断技术。顾名思义，液体活检是利用患者血液、胸腔/腹腔/心包腔积液、脑脊液、尿液、唾液等样本进行检测。与组织活检相比，液体活检标本易于获取且可反复多次取样。基于细胞游离DNA（cell-free DNA, cfDNA）、循环肿瘤DNA（circulating tumor DNA, ctDNA）、循环肿瘤细胞（circulating tumor cells, CTCs）、细胞外囊泡（extracellular vesicles, EVs）、信使RNA（messenger RNA, mRNA）、外泌体等样本的液体活检不仅能揭示肺癌基因全貌，而且可以监测肺癌基因的动态变化^[11-18]^。利用外周血提取ctDNA是目前相对成熟的液体活检技术，由于血液样本易于采集，已成为检测基因突变的常规标本，尤其是用于监测治疗反应和评估耐药机制。但是血浆中ctDNA的含量极为有限，ctDNA的提取和检测仍具挑战^[18-20]^。有研究探索其他含有ctDNA的体液，以作为液体活检样本的潜在替代品^[21]^。恶性胸腔积液（malignant pleural effusion, MPE）中富含肿瘤细胞，且胸腔积液样本易于采集。2005年有学者报道了利用MPE作为1例肺癌患者液体活检样本检测到表皮生长因子受体（epidermal growth factor receptor, *EGFR*）突变的病例报告^[22]^，开辟了研究以MPE作为液体活检样本的新道路。本文对液体活检在肺癌伴MPE诊断与治疗中的研究进展进行系统综述。

## 1 恶性胸腔积液

MPE是指经病理学证实存在脱落的肿瘤细胞或胸膜活检组织发现肿瘤细胞的胸水样本。MPE积聚在脏层胸膜和壁层胸膜之间，由肿瘤细胞侵袭或转移至胸膜，影响胸水的滤出或吸收所致^[23, 24]^。MPE是晚期恶性肿瘤的常见并发症，肺癌是其最常见的病因，约占30%^[25, 26]^。约15%的肺癌患者在初诊时就已出现MPE，而在疾病发展过程中，近40%的患者将发生MPE^[27]^。

伴随着精准医学时代的到来，肺癌的治疗由传统的化疗逐渐向分子靶向和免疫治疗发展。酪氨酸激酶抑制剂（tyrosine kinase inhibitor, TKI）小分子靶向治疗是驱动基因阳性晚期非小细胞肺癌（non-small cell lung cancer, NSCLC）的一线标准治疗，而检测肿瘤驱动基因是制定治疗决策的前提^[28]^。与组织活检相比，MPE易于收集且富含大量肿瘤细胞。

有研究^[26]^证实肺腺癌伴MPE患者的胸水样本所检测到的*EGFR*突变率高于手术切除的肺腺癌组织样本（68.4% *vs* 50.5%）。Wang等^[29]^检测了295例肺腺癌患者MPE样本，其中92例具有匹配的组织样本，研究发现，MPE上清液样本中*EGFR*突变率为39.3%，与组织样本的一致性达87.1%；以组织为金标准，MPE上清液样本检测*EGFR*突变的敏感性和特异性分别为71.4%和96.5%。同样，胸水样本能准确检测*EGFR*突变亚型，有研究利用二代测序（next generation sequencing, NGS）技术，证明了在胸水中所检测到的*EGFR*敏感突变的丰度（突变丰度是指在该基因位点所有的等位基因中，突变等位基因所占的比例）均要高于血液样本中的突变丰度^[30]^。此外，在靶向治疗过程中尚未出现影像学进展时，可以利用聚合酶链反应（polymerase chain reaction, PCR）或NGS等技术在ctDNA中检测到*EGFR* T790M突变，有助于进行早期临床干预^[31-33]^。MPE是提供*EGFR*突变信息的可靠的液体活检样本，对指导晚期NSCLC靶向治疗的药物选择具有重要的临床价值。

## 2 MPE中cfDNA的应用价值

cfDNA是正常细胞和癌细胞在发生坏死和凋亡过程中释放到血液循环中小片段的DNA，而由肿瘤原发灶或转移灶释放的这部分则称为ctDNA，ctDNA能够反映肿瘤的遗传特征^[12, 18, 34]^。ctDNA不仅可用于NSCLC的早期筛查和诊断，也能用于指导治疗及评估预后和复发^[35, 36]^。与从肿瘤组织中分离出的DNA相比，血浆ctDNA具有较高的敏感性和特异性（分别为88%和100%），是检测*EGFR*突变的可靠方法^[37]^。除了能在血浆中得以分离，ctDNA也存在于胸水、腹水及其他体液中^[38]^。Kimura等^[39]^研究表明，胸腔积液中的cfDNA可检测出*EGFR*突变，且与EGFR-TKI治疗的疗效相关。Wang等^[29]^将从MPE上清液中提取DNA检测出的*EGFR*突变的晚期NSCLC患者进行EGFR-TKI治疗，发现其客观缓解率（objective response rate, ORR）为57.6%，无进展生存期（progression free survival, PFS）为8.9个月，总生存期（overall survival, OS）为29.8个月，而组织和血浆中检测出*EGFR*突变的患者，其ORR分别为56.0%和47.4%，PFS分别为9.0个月和7.7个月，OS分别为25.9个月和25.3个月。MPE上清液还可检测间变性淋巴瘤激酶（anaplastic lymphoma kinase, *ALK*）和肉瘤致癌因子-受体酪氨酸激酶（C-ros oncogene 1, *ROS1*）融合基因。Yao等^[40]^研究了16例MPE中检测出*ALK*或*ROS1*融合基因并接受靶向治疗的晚期NSCLC患者，发现8例患者获得部分缓解（partial response, PR），4例疾病稳定（stable disease, SD），4例疾病进展（progressive disease, PD）。此外，与配对的细胞块及血浆样本检测相比，胸水上清液中能检测出更高的基因突变丰度，能提供更为全面的基因信息^[20]^。Yu等^[41]^发现MPE上清液中的长片段cfDNA会引入低丰度的与癌症不相关的变体，推荐在临床实践中，直接检测总cfDNA，而不是片段化处理后再检测。总之，从MPE中提取cfDNA用于检测NSCLC的基因突变是可行的，MPE是在肿瘤组织不可获取时检测基因突变的有效替代物，MPE的检测结果可用于指导临床治疗决策^[40, 42-46]^（表 1）。

**表 1:** 液体活检在恶性胸腔积液中的研究汇总 Clinical studies of liquid biopsy in malignant pleural effusion

Study	Sample type of MPE	Number of patients	Driver gene	Sensitivity (%)	Specificity (%)
Liu, 2013^[43]^	Cell block	11	*EGFR*	81.8	80
	Supernatant	11	*EGFR*	63.6	100
	Plasma	40	*EGFR*	67.5	100
Yeo, 2013^[42]^	Supernatant	22	*EGFR*	89.0	100
Liu, 2014^[44]^	Cell block	16	*EGFR*	87.5	75
	Supernatant	19	*EGFR*	84.2	90.9
Wang, 2019^[29]^	Supernatant	92	*EGFR*	71.4	96.5
Yao, 2019^[40]^	Cell block in treated patients	69	*EGFR*	77.6	83.8
	Cell block in untreated patients	59	*EGER*	48.8	71.9
	Cell block in treated patients	12	*ALK*	82.4	86.7
	Cell block in untreated patients	9	*ALK*	46.9	62.7
	Cell block in treated patients	3	*ROS1*	82.7	89.4
	Cell block in untreated patients	2	*ROS1*	52.6	63.1
Son, 2020^[46]^	Cell block	41	*EGFR*	93.3	96.2
	Supernatant	67	*EGFR*	84.4	97.1
	Plasma	31	*EGFR*	70.6	100
EGFR: epidermal growth factor receptor; ALK: anaplastic lymphoma kinase; ROS1: C-ros oncogene 1.					

## 3 MPE中EVs的应用价值

EVs是活细胞以旁分泌的形式释放到微环境中的膜性小囊泡，包含各类蛋白质、核酸（如DNA、miRNA、mRNA等）以及脂类和细胞代谢物等，大小在50 nm-150 nm的EVs则称为外泌体，直径在50 nm-1, 000 nm的则称为微囊泡^[47, 48]^。不同来源的EVs具有不同的功能，肿瘤细胞来源的EVs具有改变肿瘤微环境的能力，能促进肿瘤的侵袭和转移^[49-51]^。EVs能从包括胸腔积液在内的大多数体液中提取分离，来源于EVs的DNA非常适合于*EGFR*基因分型以及T790M耐药突变的检测。Lee等^[52]^比较了MPE中cfDNA与MPE中EVs来源的DNA对*EGFR*基因的检出率，发现EVs来源的DNA对*EGFR*的检出率较cfDNA更高，其检出率分别为100%（19/19）和89%（17/19）；EVs来源的DNA对EGFR-TKI治疗耐药后继发性T790M突变的检出率较cfDNA同样更高，分别为72.2%（13/18）和61.1%（11/18）。此外，来自于胸腔积液的EVs与来自于原发肺癌细胞的EVs具有相似的生化特性，其中的蛋白同样高度富集，与肺癌的发生发展关系密切。Park等从NSCLC患者的MPE中提取的EVs发现，EVs包含了912种不同的蛋白，其中EGFR蛋白、ras家族蛋白、Src酪氨酸激酶、癌胚抗原相关细胞黏附分子6（carcinoembryonic antigen-related cell adhesion molecule 6, CEACAM6）和胞质无机焦磷酸酶等可作为诊断肺癌的新型生物标志物^[38, 52-54]^。

## 4 MPE中miRNA的应用价值

如前所述，EVs不仅包含了多种肺癌诊断的蛋白标志物，也包含了miRNA在内的多种核酸。miRNA是一种稳定性好的非编码单链小RNA，介导RNA转录，调节基因表达，调控肿瘤的进展和转移，同样在MPE的发展过程中起着关键作用^[55, 56]^。miRNA难以被RNA酶降解，且在各类RNA中含量最高，能在包括肺癌在内的大多数肿瘤中表达，是诊断肺癌的新型生物标志物^[57-59]^。Khandelwal等^[60]^报道，在NSCLC中，microRNA‐590‐5p通过调节STAT3通路发挥肿瘤抑制作用，可以作为诊断NSCLC和评估预后的生物标志物，并可能成为治疗NSCLC的潜在靶点。miRNA除了在外周血中循环，在NSCLC患者的胸水中也能检出^[57]^，用于良恶性胸腔积液的鉴别诊断。相较于良性胸腔积液，miRNA-134、miRNA-22、miRNA-198和miRNA-185在肺腺癌的MPE中显著低表达，而miRNA-195-5p、miRNA-182-5p、miRNA-34a-5p和miRNA-210则显著高表达^[55, 61-63]^。除了鉴别良恶性胸腔积液外，miRNA也能用于协助诊断NSCLC。miRNA-200家族（miRNA-200b、miRNA-200c、miRNA-141）、miRNA-375、miRNA-429、miRNA-483-5p、miRNA-200a-3p、miRNA-203a-3p、miRNA-205-5p等在肺腺癌患者的胸水中显著增高，对肺腺癌具有较好的诊断效能^[56, 64]^。此外，来源于MPE的miRNA可用于指导NSCLC患者的治疗，也可用于其预后的评估。其中，miRNA-93通过血管生成素2（angiopoietin 2, Ang2）可抑制血管和淋巴管的生成，miRNA-93/Ang2可能是肿瘤治疗的潜在靶点。接受EGFR-TKI治疗的NSCLC患者中，miRNA-200c-3p高表达患者的PFS显著长于低表达患者，缺乏miRNA-200c-3p表达的患者则耐药增加；Wang等^[65-69]^分析了184例NSCLC患者的MPE样本，发现胸水中miRNA-141、miRNA-93、miRNA-134、miRNA-151和miRNA-345的低表达与患者较短的总生存期显著相关。

## 5 血液样本与MPE样本的比较

尽管目前临床上用于液体活检的样本仍主要是血液样本，但血液样本中ctDNA的含量极为有限，越来越多的研究转向对胸水样本的探索。研究发现，MPE上清液中的总cfDNA浓度显著高于血浆，其中位值分别为278.1 ng/mL和20.4 ng/mL。此外，MPE上清液中的ctDNA检测到体细胞突变的频率比MPE沉淀物和血浆样本更高，分别为98.4%（62/63）、90.5%（57/63）和87%（55/63），*EGFR*突变检出率也更高^[70]^。Wang等^[29]^研究发现，MPE上清液中*EGFR*的突变检出率为39.3%（116/295），而血浆中突变检出率仅为27.4%（68/248）。Guo等^[20]^研究发现，对于肺癌相关的168个基因的检出率，MPE上清液的检出率高于血浆样本，分别为87%（134/154）、83.1%（128/154），尽管无统计学差异，但MPE上清液的中位最高突变丰度（maximum allelic fraction, maxAF）却显著高于血浆样本，分别为20.3%和1.13%。Tong等^[70]^发现，以组织样本所检出的驱动基因突变为金标准，胸水来源的cfDNA中驱动基因突变的检出率为93%（27/29），而血浆cfDNA中的检出率仅为62%。胸水样本中ctDNA检测*EGFR*基因突变的敏感性和准确性分别为75.76%（25/33）和80.0%（32/40），均显著高于血液样本，血液样本分别为48.48%（16/33）和57.50%（23/40）^[71]^。Son等^[46]^研究发现，MPE上清液检测*EGFR*的敏感性为84.4%，特异性为97.1%，而血浆样本检测*EGFR*的敏感性和特异性分别为70.6%和100%；MPE上清液对*EGFR*外显子19缺失突变检测的敏感性和特异性分别为93.8%和100%，而血浆检测分别为75.0%和100%；对*EGFR*-L858R突变，MPE上清液检测的敏感性和特异性分别为73.3%和98.1%，而血浆检测分别为60.0%和100%。此外，Hummelink等^[45]^报道胸水上清液cfDNA可用于检测*EGFR*耐药突变，用于指导EGFR-TKI耐药后的治疗策略。综上，胸水在检测肿瘤驱动基因突变方面优于血液，可以提供有价值的临床信息，是基因检测更可靠的样本来源^[72]^。

## 6 总结

相比于血液样本，MPE中富含肿瘤细胞和肿瘤DNA，是检测肺癌基因信息极佳的样本来源，尤其是胸水上清液。MPE中所含的cfDNA、外泌体和miRNA等循环标志物的检测，能协助鉴别良恶性胸腔积液，实现肿瘤的分子分型，发现肿瘤的耐药突变，指导NSCLC精准靶向治疗，探索潜在的新型治疗靶点，并评估患者的预后。在EGFR-TKI治疗过程中，对MPE样本进行检测，能早期发现疾病的进展，从而实现早期干预，改善患者预后，降低患者的死亡率。
